# The Effect of Steel Beam Elastic Restraint on the Critical Moment of Lateral Torsional Buckling

**DOI:** 10.3390/ma15041275

**Published:** 2022-02-09

**Authors:** Rafał Piotrowski, Andrzej Szychowski

**Affiliations:** Faculty of Civil Engineering and Architecture, Kielce University of Technology, 25-314 Kielce, Poland; aszychow@tu.kielce.pl

**Keywords:** critical moment of lateral torsional buckling, elastic supports, elastic restraint against warping, elastic restraint against rotation in the beam bending plane

## Abstract

This paper reports the results of the next stage of the authors’ investigations into the effect of the elastic action of support nodes on the lateral torsional buckling of steel beams with a bisymmetric I-section. The analysis took into account beam elastic restraint against warping and against rotation in the bending plane. Such beams are found in building frames or frame structures. Taking into account the support conditions mentioned above allows for more effective design of such elements, compared with the boundary conditions of fork support, commonly adopted by designers. The entire range of variation in node rigidity was considered in the study, namely from complete freedom of warping to complete restraint, and from complete freedom of rotation relative to the stronger axis of the cross section (free support) to complete blockage (full fixity). The beams were conservatively assumed to be freely supported against lateral rotation, i.e., rotation in the lateral torsional buckling plane. Calculations were performed for various values of the indexes of fixity against warping and against rotation in the beam bending plane. In the study, formulas for the critical moment of bilaterally fixed beams were developed. Also, approximate formulas were devised for elastic restraint in the support nodes. The formulas concerned the most frequent loading variants applied to single-span beams. The critical moments determined in the study were compared, with values obtained using LTBeamN software (FEM). Good compliance of results was observed. The derived formulas are useful for the engineering design of this type of structures. The designs are based on a more accurate calculation model, which, at the same time offers simplicity of calculation.

## 1. Introduction

Lateral torsional buckling (LTB) of steel beams commonly used in general or industrial construction is an issue that has been examined by researchers for many years. A vast majority of publications focus on fork-supported beams, whereas real building structures generally have more complex boundary conditions. The reason is that the model of fork support allows for the use of simple functions, usually trigonometric ones, which specify beam displacements caused by the LTB phenomenon. Such an approach facilitates an analysis of other parameters that affect the LTB critical moment. Idealised boundary conditions of that type were taken into account to investigate the effect of the following: (a) the distribution of the bending moment over the beam length, e.g., [1,2,3,4,5,6], (b) the coordinates of the points of transverse load application over the height of the cross section, e.g., [1,7,8,9,10], (c) discrete (point-based) restraint against displacement and/or the cross sections’ torsion over of the beam length, e.g., [11,12,13,14,15,16,17,18], (d) LTB of monosymmetric cross sections [2,4,8,19], (e) the use of complete and incomplete (inserted) end plates [20,21,22], (f) coped beams [20,21,23,24,25,26,27], effect on the LTB critical moment, (g) modification of the energy equation leading to a nonlinear analysis of eigenvalue problem [28], and (h) interaction between buckling and LTB of beam columns [29,30,31,32].

For actual frames, framework, or grate structures, the fork support adopted in the computational model is considered a conservative approach, although in some cases of coped beams, a nodal element is weakened relative to the fork support [20,21,22,23,24,25,27]. The issue cannot be overlooked because oversimplification of the mounting node connection may lead to a considerable reduction in structural resistance, resulting from the condition of spatial loss of stability.

Contemporary design methods aim to provide the most accurate representation of the structural element behaviour in the computational model. Consequently, it is possible to take account of the reserves of LTB resistance of those beams, for which the boundary conditions are stronger compared with the theoretical fork support. In this way, the structural reliability is better accounted for because it is not based on unknown resistance reserves, but on objective measures.

This approach harmonises with the sustainable development principles. The structural system behaviour, which is decisive for structural reliability, is described in the computational models. Consequently, they need to be as accurate as possible. The created resistance reserve is measurable. It is not hidden in unknown resistance reserves resulting from the simplification of the boundary conditions of the member under examination. This approach allows for a more optimal design of steel structures. Taking into account the potential reserves of structure resistance puts a demand on the diligence of the designer, who needs to employ adequate computational models. Consequently, it is important to have an option of verifying computations with the use of simplified analytical methods. The dual approach to the reliability of computational procedures improves the safety of buildings already at the design stage.

In contemporary steel structures, including frames and frameworks or grates, complex boundary conditions are usually found. They are related to the following: (1) the beam elastic restraint against rotation relative to the higher rigidity axis of the cross section (the so-called major axis), (2) elastic restraint against warping in nodes, and (3) elastic restraint against rotation in the LTB plane, which particularly refers to grate structures.

In order to enhance beam resistance to LTB, systems of support ribs for beams (spandrel beams) were developed. They were meant to increase the deplanation rigidity of support cross sections [33,34,35,36,37,38]. Methods for analysing beams discretely braced along their span were discussed in [39,40,41,42]. For example, [39,40,41,42,43] investigated the critical resistance of single-span or multispan beams, and beams with cantilevers, ribbed in support cross sections and discretely braced over their length. It was indicated that ribs (especially closed ones) and discrete elastic bracings considerably affect the values of critical loads. The authors of [20,23,33,34,36,37,43,44,45,46], analysed the impact of elastic restraint against warping of support cross sections for single-span beams with bisymmetric cross sections. The impact of elastic restraint against warping of the cantilever beam free end on its critical resistance caused by the LTB condition was examined in [47]. In addition, the impact of flat ribs on the critical moment of a beam with a convergent web was investigated in [48].

In [49], the authors analysed the impact of the elastic restraint of beam warping in the support nodes on the LTB critical moment. A constant or linearly varying distribution of the bending moment was considered. Formulas for the index of elastic warping restraint *k_w_* and the coefficient C1, the value of which depends on the bending moment distribution and the rigidity of the elastic warping restraint *c_w_,* were proposed. Beam critical resistances were determined for different end plate thicknesses. The resultant critical moments were compared with the FEM results (LTBeamN, ANSYS) and with the values obtained from the approximate formulas derived in [50]. A good congruence of results was observed.

In all the studies quoted above, the deplanation function of a thin-walled section was developed in accordance with the Vlasov theory [51]. Such an approach seems sufficient in the case of engineering calculations, e.g., [2,6,7,10,14,18,20,23,50,52] in the analysis of beams with a homogeneous steel cross section, especially hot-rolled ones. That can be seen in European standards for the design of metal structures [53,54,55]. It was assumed in the standards that the “shear lag” effect should be primarily considered for those plate girder sections or cold-formed elements, in which a substantial width of the cantilever or spanning chords (e.g., decks in steel bridges) were found. Detailed guidelines are given in Section 3 of the standard [55].

A more general formulation of the warping function for cross sections of structural members is given in [56]. The formulation is particularly applicable to composite structures. The paper proposed a new theory termed Generalized Warping Beam Theory (GWBT), which takes into account the effect of nonuniform warping of the cross section. That was done using a single, independent parameter for each warping type. Shear warping in each direction, and also primary as well as secondary torsional warping, were considered. This approach accounted for shear lag in both the element bending and in torsion. Many examples of computations were included to confirm the effectiveness of the method.

The effect of the elastic action of nodes with respect to the LTB critical moment in beams, analysed in accordance with the thin-walled bar theory [51], was investigated by Giżejowski [23] as well as Giżejowski and Bródka [57]. The solution [23], a modification of the Lindner proposal [20] and the extension of the formula specified in ENV 1993-1-1 [58], used the concept of buckling length coefficients. Like for axially compressed elements, the coefficients were applied to flexural buckling (relative to the cross section minor axis) and torsional buckling (relative to the bar longitudinal axis). The coefficients were determined in the same way as for the buckling of braced frame columns. It was concluded that taking into account the additional rigidity of complete end plates combined with other elements of a framework structure contributed to an increase in the LTB critical moment.

According to the authors’ knowledge, apart from [23,57], the literature on the subject does not offer unambiguous analytical formulas for the LTB critical moment, which would simultaneously take account of the effect of elastic restraint against warping and elastic restraint against rotation relative to the major axis in support cross sections.

Obviously, such calculations can be performed using the finite element method, e.g., LTBeam or LTBeamN software employing finite bar elements, or by utilising more advanced 3D modelling, e.g., Abaqus software with shell or solid elements. However, it should be emphasised that, in order to improve the safety of structures already at the design stage, FEM calculations should be verified by at least a simplified analytical estimation. Such approximate formulas could also facilitate more advanced preliminary design. As regards basic static schemes, the formulas can be applied at the principal design stage. An expert analysis revealed cases in which designers less experienced in FEM modelling made mistakes when constructing a numerical model (e.g., modelling the boundary conditions in the beam support nodes). Then, relatively simple approximate formulas make it possible to correct calculations.

A good example of such an approach is offered in [59]. The authors developed approximate analytical formulas to determine local buckling stress for a large group of hot-rolled sections under complex load conditions. The calculations can also be performed using FEM software, e.g., Abaqus or FSM, e.g., CUFSM. However, the possibility of verifying the results of numerical calculations with relatively simple approximate formulas encouraged the authors to carry out extensive research in this field.

As already mentioned, many studies show that, in an analysis of LTB of beams with fork support, in order to approximate the function of beam displacements (angle of twist, lateral deflection), the researchers usually use trigonometric functions, which provide a good approximation of the critical resistance. However, in the analysis of elastically restrained beams, this approach causes difficulties when describing the degree of elastic fixity of beam support cross sections. Therefore, in their previous studies [50,52,60], the authors utilised an alternative method for the description of beam displacements upon a loss of stability, namely employing power polynomials with a simple physical (static) interpretation. As indicated before, this approach facilitates the description of the torsion angle function and beam lateral deflection when the conditions of its elastic restraint against warping and lateral rotation in the support nodes are taken into account. A detailed discussion of the approach is given in [50,52]. Power polynomials proved successful in a stability analysis of thin-walled members [61], and also in studies into local buckling of the thin-walled elements with open cross sections subjected to warping torsion [62].

In the first stage of their research [60], the authors verified that it is correct to use power polynomials when approximating the function of the twist angle (*φ*) and lateral deflection (*u*) of a beam with fork support. Computational algorithms for M_LTB_ were developed using Mathematica^®^ software, and approximate formulas were provided to estimate the beam critical resistance. The beam loading schemes most commonly found in engineering practice were taken into consideration. A comparative analysis of the results obtained [60] against the values generated by the LTBeam software [63] (FEM) and by formulas available in the literature [1,58] indicated the correctness of the applied solution.

The next stage of research [52] took into account the elastic restraint of warping in the support cross sections of a beam simply supported in the bending plane. In this case, the innovative “coupling” of the power polynomials, which describe the deflection function of the simply supported beam and the deflection function of a bilaterally fixed beam, was used to approximate the twist angle function (*φ*). Computational algorithms were developed for M_LTB,EL_ (Mathematica^®^), and approximate formulas were devised. Elastic restraint of warping was included by means of the fixity index *κ_ω_*. A comparative analysis of the results obtained [52] and the values provided by LTBeam [63] and Abaqus (FEM) software, as well as formulas available in the literature [34], was performed. The analysis indicated the effectiveness of the applied solution.

The issues related to the interaction of beam elastic restraint against warping and against lateral rotation (in the LTB plane) were discussed in [50]. The beam was assumed to be simply supported in the bending plane. Such elements are found, e.g., in certain types of steel grates. In the analytical solution to the problem, the coupling of polynomials proposed in [52] was applied to approximate both the function of the twist angle (*φ*) and the function of lateral deflection (*u*). Such an approach allowed for alterations in the form of the function of displacements (the angle of twist and lateral deflection), depending on the degree of elastic restraint against warping and against lateral rotation on the supports. Computational algorithms were developed for M_LTB,EL,2_ (Mathematica^®^), and approximate formulas were proposed. Elastic restraint against warping and against lateral rotation was included by introducing fixity indices *κ_ω_* and *κ_u_*, respectively. A comparative analysis of the results produced [50] and the values obtained through LTBeam software [63] (FEM) showed it was correct to apply the solution mentioned above.

From a technical standpoint, another issue that considerably affects the LTB critical moment in beams is the elastic restraint against rotation in the bending plane (i.e., rotation relative to the major axis of the cross section) in the support nodes.

The application range of approximate formulas proposed in [50,52,60] can be extended. This study is a positive step in this direction. The study deals with the LTB of single-span beams with a bisymmetric I-section, which are elastically restrained in the support nodes against warping and against rotation in the bending plane. Elements of this class are found in frames or framework structures (e.g., in supporting structures for technological devices of industrial buildings). A conservative assumption was made that the conditions of hinged support occur during lateral rotation on supports (in the LTB plane). Elastic restraint in the LTB plane is considered to be important in grates with complex nodes, while in frames and framework structures it is less significant. The simultaneous effect of elastic restraint against warping, rotation relative to the major axis, and relative to the minor axis of the cross section in the support nodes will be the subject of the authors’ further research.

In this study, formulas were derived for the LTB critical moment (*M_cr,u_*) of bilaterally fixed beams (in the bending plane) for any degree of elastic restraint against warping in the support nodes. Formulas for *M_cr,o_* of simply supported beams were developed in [52]. Based on the limit values of critical moments for simply supported *M_cr,o_* and fully fixed beams *M_cr,u_*, approximate formulas for *M_cr_* were devised for beams elastically restrained in the support nodes. Detailed calculations were performed for beams with various indices of fixity against warping *κ_ω_* and against rotation in the bending plane *κ_ν_*. Symmetrical boundary conditions relative to the midspan of a beam were adopted. The results obtained were compared with those from FEM (LTBeamN).

In this study, the following assumptions were made: (1) over its length, the single-span beam has a constant, bisymmetrical hot-rolled I-section, or its welded equivalent, (2) boundary conditions, symmetrical with respect to the beam midspan, are found, (3) three load distributions, most common in engineering practice, are considered, (4) the conditions of the beam elastic support concern the following: (a) restraint of rotation with respect to the major axis of the cross section, (b) limitation of the cross section warping (warping function in accordance with [51]).

Compared with the solutions discussed in the literature, this study offers a novel approach, which involves the following:

(i) Approximate formulas were derived for the LTB critical moment (*M_cr,u_*) of steel beams with bisymmetrical cross section that are bilaterally fixed against bending (*M_y_*) and elastically restrained against warping. The LTB critical moment represents the upper limit of the critical resistance in the elastic range.
(ii) Approximate formulas were derived for *M_cr_*, for any degree of elastic restraint against rotation about the section major axis, and against warping at the support nodes. That was done based on the indexes of fixity that are independent of one another.
(iii) A solution was obtained that allows for a more accurate representation of the actual LTB behaviour of a steel beam using a relatively simple analytical model (cf. calculation example in Section 5.4).

## 2. Beam Elastic Restraint against Warping and against Rotation in Its Bending Plane

**Figure 1:**
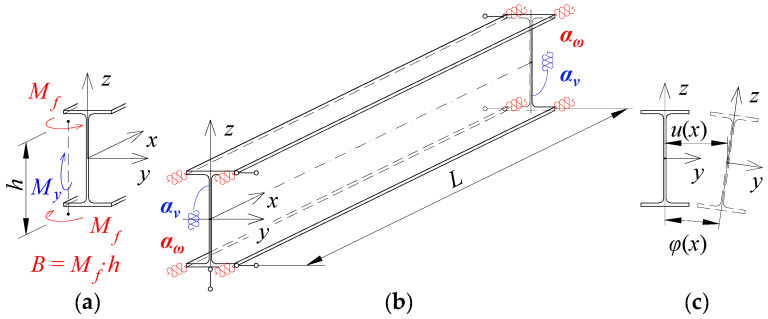
Static scheme of a beam: (**a**) the bimoment *B* and moment *M_y_* on a support, (**b**) elastic restraint against warping (*α_ω_*) and against rotation in the bending plane of the beam (*α_ν_*), (**c**) angle of twist *φ*(*x*) and lateral deflection *u*(*x*) of the beam.

The static scheme, adopted in this study, of a beam elastically restrained against warping and against rotation in its bending plane in the support nodes is shown in Figure 1.

The degree of elastic restraint of the beam in the support nodes (Figure 1) was accounted for by the use of dimensionless indices of fixity against warping *κ_ω_* [50,52] and against rotation in the bending plane *κ_ν_*.

As regards elastic restraint against warping, the fixity index *κ_ω_* has the following form [50,52]:

(1) $$\kappa_{\omega }=\frac{\alpha_{\omega }L}{2EI_{\omega }+\alpha_{\omega }L},$$

where *L*—beam span, *E*—Young’s modulus, *I_ω_*—warping constant, and *α_ω_*—rigidity of elastic restraint against warping [35,37,50,52] according to:

(2) $$\alpha_{\omega }=-\frac{B}{d\varphi /dx},$$

where *B*—bimoment at the support point of the beam, *φ*—angle of twist, and *dφ/dx*—warping of the section.

The index of elastic fixity against warping changes is from *κ_ω_* = 0 for complete warping freedom to *κ_ω_* = 1 for full prevention of warping.

The *κ_ν_* index of elastic fixity of the beam support section against rotation in the bending plane (i.e., against rotation relative to the major axis of the cross section) is expressed by the equation:

(3) $$\kappa_{\nu }=\frac{\alpha_{\nu }L}{4EI_{y}+\alpha_{\nu }L},$$

where *I_y_*—second moment of inertia in bending about the *y*-axis; *α_ν_*—rigidity of elastic restraint against rotation in the beam bending plane according to the equation:

(4) $$\alpha_{\nu }=\frac{M_{y}}{d\nu /dx},$$

where *M_y_*—bending moment relative to the major axis of the support cross section, *ν*—vertical deflection, and *dν/dx*—rotation relative to the *y* axis in the support cross section.

The index of fixity against rotation in the bending plane of the beam ranges from *κ_ν_* = 0 for complete freedom of rotation (hinge support) to *κ_ν_* = 1 for complete prevention of rotation (fixity).

The rigidities of elastic restraint against warping *α_ω_* (Equation (2)) and against rotation *α_ν_* (Equation (4)) can be expressed as a function of the fixity indices (*κ_ω_*, *κ_ν_*) according to the equations:

(5) $$\alpha_{\omega }=\frac{2\kappa_{\omega }EI_{\omega }}{\left(1-\kappa_{\omega }\right)L};\;\;\;\;\;\;\;\;\;\;\;\;\;\;\;\alpha_{\nu }=\frac{4\kappa_{\nu }EI_{y}}{\left(1-\kappa_{\nu }\right)L}.$$

## 3. LTB Critical Moment of a Fixed Beam

### 3.1. Function of the Twist Angle

A program for numerical calculations (M_LTB,EL_) was proposed in [52]. With the program, it is possible to determine the LTB critical moment of a beam, which in its support nodes is simply supported in the bending plane, and elastically restrained against warping. In M_LTB,EL_ software, the function of the beam twist angle (*φ*) was approximated using the innovative “coupling” of power polynomials. This was done according to the equation [52]:

(6) $$\varphi \left(x\right)=\sum_{i=1}^{3}a_{i}\left(\left(1-\kappa_{\omega }\right)\cdot W_{Pi}+\kappa_{\omega }\cdot W_{Ui}\right),$$

where *a_i_*—free parameters of the twist angle function, *κ_ω_*—elastic restraint index according to Equation (1); *W_Pi_*—polynomials describing the deflection function of a simply supported beam; and *W_Ui_*—polynomials describing the deflection function of a fixed beam.

The power polynomials (*W_Pi_*, *W_Ui_*) used in [52], along with their physical interpretation, are shown in Table 1 (where *ρ* = *x*/*L*).

The deflection polynomials adopted for the twist angle function fulfil the boundary conditions for the freedom of warping *W_Pi_* (*φ* = 0, *φ*” = 0 for *x* = 0 and *x* = *L*), and for the complete prevention of warping *W_Ui_* (*φ* = 0, *φ*’ = 0 for *x* = 0 and *x* = *L*) in the support nodes, respectively.

In [50,52], it was demonstrated that the twist angle function—Equation (6) together with the polynomials (*W_Pi_* and *W_Ui_*) shown in Table 1—makes it possible to model elastic restraint against warping for any value of the fixity index from a range of 0 < *κ_ω_* < 1.

### 3.2. Determination of M_cr,u_ with the Energy Method

The elastic LTB critical moment of a bilaterally fixed (*M_cr,u_*), bisymmetric I-beam was determined using the energy method [9]. The elastic restraint against warping in the support nodes was taken into account.

The degree of elastic restraint of nodes in the beam bending plane is not a typical boundary condition for lateral–torsional buckling. However, it strongly affects the longitudinal distribution of the bending moment *M_y_*. As a result, in order to determine *M_cr,u_* for beams fixed in the bending plane and elastically restrained against warping on the supports, the work done by external forces for this type of support and load should be taken into account in the functional of the total potential energy. In such an approach, complete prevention of rotation of the beam cross section in the precritical bending plane is represented by the support moments of restraint. It should be noted that in the precritical state (*M_y_* < *M_cr_*), the bending of a beam proceeds relative to the *y*–*y* axis, while in the post-critical state (*M_y_* > *M_cr_*), due to lateral deflection and the angle of twisting after LTB, the bending of the beam is biaxial.

Boundary conditions corresponding to LTB should be separated from boundary conditions that affect the distribution of support moments *M_y_* (fixity in the bending plane). This is important in the case of elastic restraints of nodes. Such restraints occur in modern node structures, especially in framework systems, where simplified connection details are used. The separation of the boundary conditions of LTB from the static scheme of the support is also found in the LTBeamN software.

As a result, the load critical value was calculated from the equation:

(7) $$\Delta \Pi =\Delta U_{s,1}+\Delta U_{s,2}-\Delta T,$$

where Δ*U_s_*_,1_—elastic energy of beam bending and torsion, Δ*U_s_*_,2_—energy of beam elastic restraint against warping in the support nodes, and Δ*T*—work done by external forces.

The elastic energy of beam bending and torsion was determined based this equation from [9]:

(8) $$\Delta U_{s,1}=\frac{1}{2}\left(EI_{z}\int_{0}^{L}{\left(\frac{d^{2}u}{dx^{2}}\right)}^{2}dx+GI_{t}\int_{0}^{L}{\left(\frac{d\varphi }{dx}\right)}^{2}dx+EI_{\omega }\int_{0}^{L}{\left(\frac{d^{2}\varphi }{dx^{2}}\right)}^{2}dx\right),$$

where *I_z_*—second moment of inertia in bending about *z*-axis, *I_t_*—Saint Venant’s torsion constant, *G*—shear modulus, and *u*—the lateral deflection function.

The energy of elastic restraint against warping in the support nodes was determined from the following equation [52]:

(9) $$\Delta U_{s,2}=\frac{\alpha_{\omega }}{2}\left({\left(\frac{d\varphi }{dx}\right)}_{x=0}^{2}+{\left(\frac{d\varphi }{dx}\right)}_{x=L}^{2}\right).$$

The work done by external forces Δ*T* depends on the beam static scheme, and is a function of the *z_g_* coordinate of the point of transverse load application. Equations expressing the work done by external forces in the most common loading variants of bilaterally fixed beams are listed in Table 2.

Equation (8) and the equations listed in Table 2 include the function of lateral deflections (*u*) relative to the minor axis *z*–*z* (see Figure 1), and the function of the twist angle (*φ*). To be able to describe the behaviour of the beam using only one function (*φ*), Equation (10) was selected out of three equilibrium equations for a beam with LTB [9]:

(10) $$EI_{z}\frac{d^{2}u}{dx^{2}}=M_{z}.$$

Following the pathway proposed in [52], Equation (7) and the function of the twist angle—Equation (6) together with the polynomials listed in Table 1—were used to develop computational programs. The programs were designed in the environment of the Mathematica*^®^* package. M_LTB,EL,u_ software makes it possible to determine the LTB critical moments for many variants. They concern geometrical parameters of bisymmetric I-sections, schemes of bilaterally fixed beams (Table 2), any coordinate (*z_g_*) of the transverse load application point, and any value of the fixity index *κ_ω_* according to Equation (1).

Examples of the calculations performed using the M_LTB,EL,u_ software are given in Section 5.

### 3.3. Approximate Equation for M_cr,u_ in a Bilaterally Fixed Beam

In [52], based on symbolic computations, relatively simple approximate equations were developed to find the LTB critical moment for simply supported beams with a bisymmetric I-section, elastically restrained against warping on supports. That involved the use of the first (*i* = 1) term of the twist angle function (Equation (6)) (i.e., of the “coupled” polynomials *W_P_*_1_ and *W_U_*_1_ from Table 1). The results obtained showed very good agreement with the FEM findings (LTBeam).

**Figure 2:**
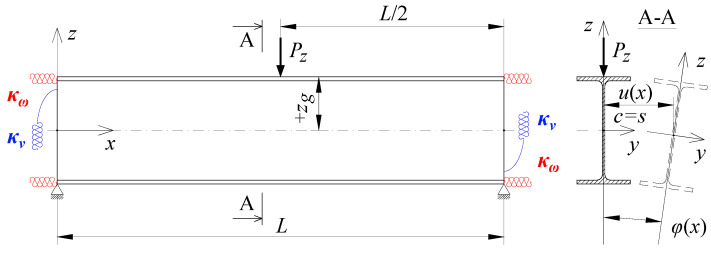
The static scheme of a beam elastically restrained in the support nodes (*κ_ω_*, *κ_ν_*) under the load of a force concentrated (*P_z_*) at the midspan.

The equation for the LTB critical moment *M_cr_* was worked out in [52]. The equation took into account the beam elastic restraint against warping (*κ_ω_*) in the support nodes, and also any ordinate (*z_g_*) of the point of transverse load application, relative to the shear centre of the cross section. The equation is as follows [52]:

(11) $$M_{cr}=\frac{-B_{1}EI_{z}z_{g}+\sqrt{EI_{z}\left(B_{3}GI_{t}L^{2}+B_{4}EI_{\omega }+B_{1}^{2}EI_{z}z_{g}^{2}\right)}}{B_{2}L^{2}},$$

where *z_g_*—ordinate of the point of transverse load application with respect to shear centre (see Figure 2) and *B*_1,_ *B*_2,_ *B*_3,_ *B*_4_—coefficients according to Table 3.

Table 3 lists the *B*_1_, *B*_2_, *B*_3_, and *B*_4_ coefficients for beams simply supported against bending *M_y_*, and the most common loading schemes [52].

The procedure adopted in this study is the same as that employed in [52]. The McrLT_fix_sym.cal.nb program was developed in the Mathematica*^®^* package to carry out symbolic transformations. Like in [50,52], the function of the twist angle was approximated only by the first (*i* = 1) term of the series (Equation (6)) using the polynomials *W_P1_* and *W_U1_* (Table 1). As a result, it was possible to devise a relatively simple approximate equation concerning the LTB critical moment for an I-beam, bilaterally fixed (for bending *M_y_*) and elastically restrained against warping. The equation was transformed into the form of Equation (11). Coefficients *B*_1_, *B*_2_, *B*_3_, and *B*_4_ for the most common variants of load application in bilaterally fixed beams are shown in Table 4.

Calculations for particular cases of critical moments for a simply supported *M_cr,o_* and fixed beam *M_cr,u_* depending on the *κ_ω_* index are provided in Section 5. The calculations were carried out using Equation (11), and the coefficients listed in Table 3 and Table 4.

## 4. Approximate Equations for the LTB Critical Moment in an Elastically Restrained Beam

In the section, the scope of approximate Equation (11) is extended to include the case of elastic restraint of a beam against rotation in the bending plane (Figure 2).

As already mentioned, the degree of elastic restraint of nodes in the beam bending plane is not a typical boundary condition for lateral–torsional buckling. However, it strongly affects the longitudinal distribution of the bending moment *M_y_*. Therefore, it indirectly influences *M_cr_*, which can be used when constructing the approximate equations.

Additionally, in the engineering computational model, the design value of the critical moment *M_cr_* was assumed to be associated with the extreme value of the moment *M_y_* over the length of the beam, regardless of the sign of this moment. Because of this, for a simply supported beam under a uniform load, the maximum bending moment *M_y,max_* = *ql^2^*/8 occurs at the midspan and causes compression of the top flange. However, in the case of a fully bilaterally fixed beam under the same load, *M_y,max_* = *ql^2^*/12 occurs on the support and causes maximum compression of the bottom flange. That further complicates the solution to the problem. It happens because a change in the place of the extreme moment occurrence, associated with the engineering interpretation of the critical moment, leads to a kink (bend in the curve) in the *M_cr_*(*κ_v_*) graph. A similar situation is also found for a triangularly distributed load, where, for a simply supported beam, the maximum bending moment *M_y,max_* = *ql^2^*/(9√3) occurs at 1/√3 of the span, causing compression of the top flange. With respect to a fully bilaterally fixed beam under the same load, *M_y,max_* = *ql^2^*/20 occurs on the support under maximum load, resulting in the maximum compression of the bottom flange.

**Figure 3:**
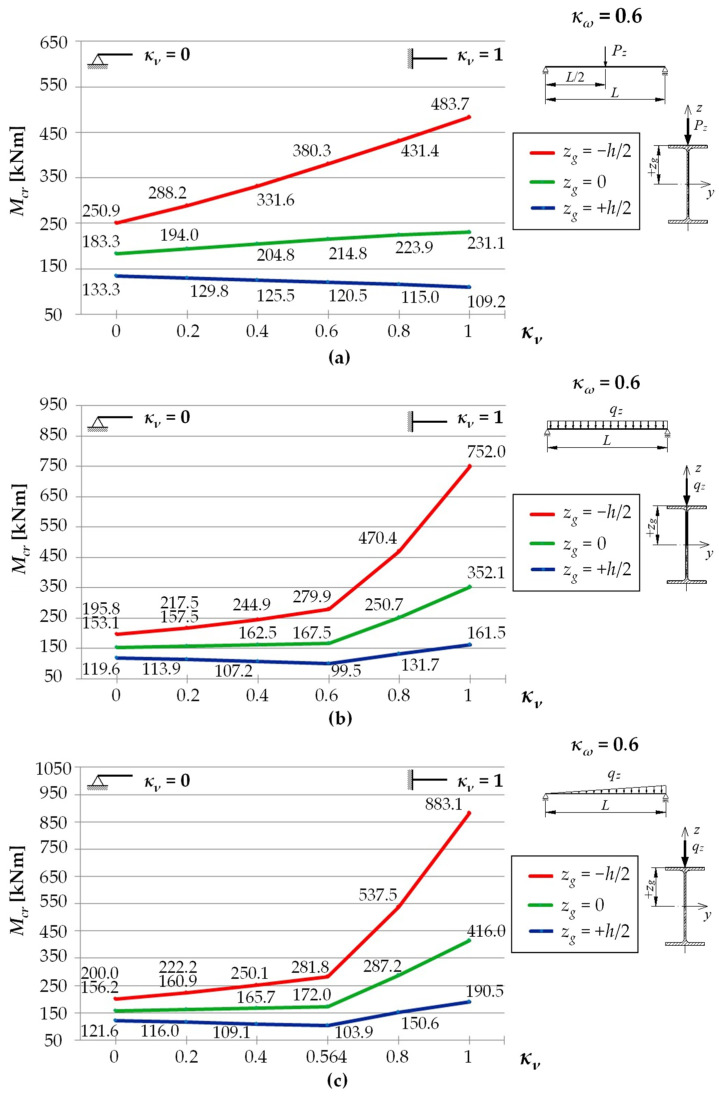
Trends in *M_cr_* variation according to LTBeamN software as a function of the fixity index *κ_ν_*: (**a**) beam under the concentrated force load at the midspan, (**b**) beam under a uniform load, (**c**) beam under a triangularly distributed load.

Figure 3 shows exemplary trends in *M_cr_*(*κ_ν_*) variation for an IPE300 beam with a span of *L* = 5 m, under the load of: (a) a concentrated force, (b) a uniform load, (c) a triangularly distributed load, for the *κ_ω_* index = 0.6, determined through LTBeamN software (FEM).

The bends in the curves for cases (b) and (c), observed in Figure 3, result from a change in the point of the maximum moment occurrence. This phenomenon was discussed above. In addition, when the load is applied to the top flange, within the variation range of the index 0 < *κ_ν_* < 0.6 (for a uniform load) and for 0 < *κ_ν_* < 0.564 (for a triangular load), an apparent fall in *M_cr_* is observed. That results from a change in *M_y_* distribution as a function of *κ_ν_*. It is obvious that, in each of the cases above, an increase in $\kappa_{v}$ is accompanied by an increase in the value of the respective critical resistance measured by the external load of the beam (*q_cr_*, *q_Tcr_*).

**Figure 4:**
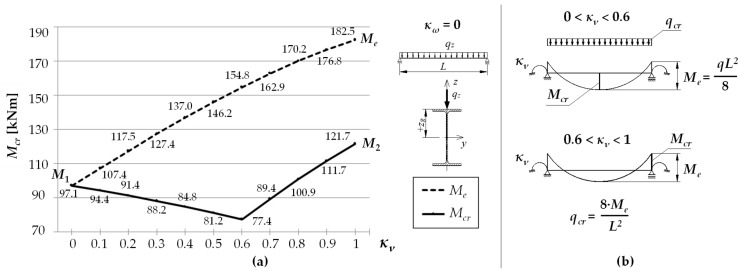
(**a**) Exemplary graph of *M_cr_*(*κ_ν_*) for IPE300 (*L* = 5 m) according to LTBeam, (**b**) definition of the equivalent moment *M_e_*.

This effect can be seen in Figure 4. The example concerned an IPE300 beam with a span of *L* = 5 m, under a uniform load at the height of the top flange, for *κ_ω_* = 0. The solid line (Figure 4a) represents a graph of the critical moment variation as a function of the *κ_ν_* index determined according to LTBeam (FEM). The broken line indicates a graph of the so-called equivalent moment *M_e_* defined in Figure 4b. The symbols *M*_1_ and *M*_2_ represent the critical moments for a simply supported (*κ_ν_* = 0) and completely fixed (*κ_ν_* = 1) beam, respectively. A kink in the graph of the *M_cr_*(*κ_ν_*) curve illustrates a change in the place of occurrence of the *M_y_* maximum absolute value. A similar effect is also observed for other values of the *κ_ω_* index from a range of (0–1).

The decrease in the value of *M_cr_* despite an increase in the *κ_ν_* index also occurs in the case of a load with a concentrated force. This applies to the entire variation range of *κ_ν_*. The reason is that, with *κ_ν_* < 1, the *M_y_* maximum value always occurs in the span (for *κ_ν_* = 1, the absolute values of the span and the support moment are equal), and the point of *M_ymax_* occurrence does not change.

Based on the analyses of the issues mentioned above, the authors decided to search for approximate equations containing the values of *M_cr_*_,*o*_ and *M_cr,u_*, which should be determined depending on the index of elastic restraint against warping (*κ_ω_*).

The approximate equation for the LTB critical moment of a beam elastically restrained in the support nodes has the following form:

(12) $$M_{cr}\left(\kappa_{\omega },\kappa_{v}\right)=M_{cr,o}\left(\kappa_{\omega },\kappa_{v}=0\right)+\left[M_{cr,u}\left(\kappa_{\omega },\kappa_{v}=1\right)-M_{cr,o}\left(\kappa_{\omega },\kappa_{v}=0\right)\right]\cdot \eta \left(\kappa_{v}\right),$$

where $M_{cr,o}\left(\kappa_{\omega },\kappa_{v}=0\right)—$the LTB critical moment for a simply supported beam and a given value of the $\kappa_{\omega }$ index, $M_{cr,u}\left(\kappa_{\omega },\kappa_{v}=1\right)—$the LTB critical moment for a fully fixed beam and a given value of the $\kappa_{\omega }$ index, $\eta \left(\kappa_{v}\right)$—the coefficient of interaction.

Equation (12) can be converted to a simplified form:

(13) $$M_{cr}=M_{o}+\left(M_{u}-M_{o}\right)\cdot \eta_{,}$$

where the following were substituted: $M_{o}=M_{cr,o}\left(\kappa_{\omega },\kappa_{v}=0\right),\;M_{u}=M_{cr,u}\left(\kappa_{\omega },\kappa_{v}=1\right),\;\eta =\eta \left(\kappa_{v}\right)$.

Adopting the above makes it easier to solve the problem, and offers a relatively simple estimation of *M_cr_* at a technically sufficient level of accuracy (±5%).

A similar concept of the so-called coefficient of interaction was employed earlier while devising approximate equations that involved the local buckling of hot-rolled sections, as discussed in [59].

Equations for the coefficient of interaction *η*(*κ_ν_*) were developed for three loading schemes of a single-span beam, which are most commonly found in practice: (a) a force concentrated at the midspan *P*, (b) a uniformly distributed load *q*, and (c) a triangularly distributed load *q_T_*. For each of the load types mentioned above, the *η* coefficients were determined for three typical coordinates of the point of transverse load application, i.e., (a) a load applied to the top flange—TF (*z_g_* = +*h*/2), (b) to the centre of gravity of the cross section—CG (*z_g_* = 0), and (c) a load applied to the bottom flange—BF (*z_g_* = −*h*/2).

The development of approximate equations for the coefficient of interaction η was preceded by numerical simulations for large sets of cases, performed using LTBeamN software. The most common hot-rolled sections, namely IPE, HEA, and HEB, were taken into consideration. On this basis, it was concluded that:

- in the case of the load of (a) a force concentrated at the midspan, (b) uniformly distributed, or (c) distributed triangularly and applied to the top flange TF, the critical load (*P_cr_*, *q_cr_*, *q_Tcr_*) can be determined as a linear combination of a critical load for a simply supported (*P_o_*, *q_o_*, *q_To_*) and fully fixed beam (*P_u_*, *q_u_*, *q_Tu_*) according to the equations:

(14) $$P_{cr}=\left(1-\kappa_{v}\right)P_{o}+\kappa_{v}P_{u}\;q_{cr}=\left(1-\kappa_{v}\right)q_{o}+\kappa_{v}q_{u}\;q_{Tcr}=\left(1-\kappa_{v}\right)q_{To}+\kappa_{v}q_{Tu}.$$

As for a uniformly or triangularly distributed load, a change in the location of the extreme value of the moment *M_y_* (identified with the critical moment *M_cr_* and expressed as a function of the *κ_ν_* index) also occurs; the coefficient of interaction depends on the limit values of *M_o_*, *M_u_*, i.e., $\eta =\eta \left(\kappa_{v},\;M_{o},\;M_{u}\right)$.

- in the case of a concentrated force load at the midspan, applied in the axis of gravity of the cross section CG, or applied to the bottom flange BF of the beam, the $\eta \left(\kappa_{v}\right)$ coefficient depends on the *κ_ν_* index in a linear manner (see Figure 3a) for various coefficients of proportionality $\eta =a_{i}\kappa_{v}$ (where: $a_{i}$—a coefficient depending on the point of the load application, CG or BF);
- in the case of a load distributed uniformly or triangularly, and applied in the axis of gravity of the cross section CG, or applied to the bottom flange BF of the beam, the best results for the $\eta \left(\kappa_{v}\right)$ coefficient were obtained when assuming its linear variation as a function of the $\kappa_{v}$ index in the form of $\eta =a_{i}\kappa_{v}+b_{i}$ (where: $a_{i},\;b_{i}$—coefficients depending on the point of load application).

The proposed approximate equations produce accuracy of the critical moment *M_cr_* estimation with an average level of ±5%. In rare cases, the error in estimation could be higher, but does not exceed ±8%. It should be indicated that *M_cr_* is used to determine the relative slenderness of a beam in lateral–torsional buckling, based on which, the reduction factor of LTB (*χ_LT_*) is determined. The accuracy of estimation of *M_cr_* with a level of even ±8% translates into the accuracy of estimation of the *χ_LT_* factor with a maximum level of ±1.5%, which is sufficient from a technical standpoint. Better accuracy of *M_cr_* estimation could be achieved, but at the cost of a considerable extension of the approximate equations.

The equations for the $\eta \left(\kappa_{v}\right)$ coefficient put forward in this study take the following form:

1. Load of a force concentrated at the midspan:

TF:

(15) $$\eta \left(\kappa_{v}\right)=\frac{2\kappa_{v}}{1+\kappa_{v}}.$$

CG:

(16) $$\eta \left(\kappa_{v}\right)=\kappa_{v}.$$

BF:

(17) $$\eta \left(\kappa_{v}\right)=0.95\kappa_{v}.$$

2. Uniformly distributed load:

TF:

(18) $$\begin{array}{l} \mathrm{for}\;a\;\mathrm{range}\;\mathrm{of}:\;0<\kappa_{v}\leq 0.6\\ \eta \left(\kappa_{v}\right)=\frac{\kappa_{v}\left[\left(2.333-0.333\kappa_{v}\right)M_{0}+\left(0.5\kappa_{v}-1.5\right)M_{u}\right]}{\left(1+\kappa_{v}\right)\left(M_{0}-M_{u}\right)};\\ \mathrm{for}\;a\;\mathrm{range}\;\mathrm{of}:\;0.6<\kappa_{v}<1\\ \eta \left(\kappa_{v}\right)=\frac{\left[1+\kappa_{v}\left(1.333\kappa_{v}-0.333\right)\right]M_{0}-2\kappa_{v}{}^{2}M_{u}}{\left(1+\kappa_{v}\right)\left(M_{0}-M_{u}\right)}. \end{array}$$

CG:

(19) $$\begin{array}{l} \mathrm{for}\;a\;\mathrm{range}\;\mathrm{of}:\;0<\kappa_{v}\leq 0.6\\ \eta \left(\kappa_{v}\right)=0.12\kappa_{v};\\ \mathrm{for}\;a\;\mathrm{range}\;\mathrm{of}:\;0.6<\kappa_{v}<1\\ \eta \left(\kappa_{v}\right)=2.28\kappa_{v}-1.32. \end{array}$$

BF:

(20) $$\begin{array}{l} \mathrm{for}\;a\;\mathrm{range}\;\mathrm{of}:\;0<\kappa_{v}\leq 0.6\\ \eta \left(\kappa_{v}\right)=0.22\kappa_{v};\\ \mathrm{for}\;a\;\mathrm{range}\;\mathrm{of}:\;0.6<\kappa_{v}<1\\ \eta \left(\kappa_{v}\right)=2.02\kappa_{v}-1.12. \end{array}$$

3. Triangularly distributed load:

TF:

(21) $$\begin{array}{l} \mathrm{for}\;a\;\mathrm{range}\;\mathrm{of}:\;0<\kappa_{v}\leq 0.564\\ \eta \left(\kappa_{v}\right)=\frac{\kappa_{v}\left[\left(2.386-0.386\kappa_{v}\right)M_{0}+\left(0.495\kappa_{v}-1.283\right)M_{u}\right]}{\left(1+\kappa_{v}\right)\left(M_{0}-M_{u}\right)};\\ \mathrm{for}\;a\;\mathrm{range}\;\mathrm{of}:\;0.564<\kappa_{v}<1\\ \eta \left(\kappa_{v}\right)=\frac{\left[1+\kappa_{v}\left(1.386\kappa_{v}-0.386\right)\right]M_{0}-1.778\kappa_{v}{}^{2}M_{u}}{\left(1+\kappa_{v}\right)\left(M_{0}-M_{u}\right)}. \end{array}$$

CG:

(22) $$\begin{array}{l} \mathrm{for}\;a\;\mathrm{range}\;\mathrm{of}:\;0<\kappa_{v}\leq 0.564\\ \eta \left(\kappa_{v}\right)=0.1\kappa_{v};\\ \mathrm{for}\;a\;\mathrm{range}\;\mathrm{of}:\;0.564<\kappa_{v}<1\\ \eta \left(\kappa_{v}\right)=2.04\kappa_{v}-1.13. \end{array}$$

BF:

(23) $$\begin{array}{l} \mathrm{for}\;a\;\mathrm{range}\;\mathrm{of}:\;0<\kappa_{v}\leq 0.564\\ \eta \left(\kappa_{v}\right)=0.19\kappa_{v};\\ \mathrm{for}\;a\;\mathrm{range}\;\mathrm{of}:\;0.564<\kappa_{v}<1\\ \eta \left(\kappa_{v}\right)=1.9\kappa_{v}-1.03. \end{array}$$

With the form of the approximate equations for the loading diagrams shown above, it is possible to create simple spreadsheets or carry out manual calculations. An example of such calculations is given in Section 5.3. It must be remembered that the values of the moments *M_o_* and *M_u_* are to be substituted into Equations (12), (13), (18) and (21) as absolute values.

## 5. Verification of Approximate Equations by FEM

### 5.1. Assumptions

Analytical calculations performed according to the approximate Equation (11) for bilaterally fixed beams, and Equation (13) for elastically restrained beams, were verified using LTBeam and LTBeamN (FEM) software.

LTBeam software [63] is based on the finite element method (FEM), in which the beam is discretised with bar elements, modified from the classical approach. Cross section warping, based on the thin-walled bar theory, constitutes an additional degree of freedom of such an element. In the nodes generated along the length of the beam, the condition of the continuity of displacements caused by the LTB phenomenon is satisfied by four degrees of freedom (lateral displacement, rotation in the LTB plane, twist of the beam along the longitudinal axis, and warping). In the LTBeam program, the critical load multiplier *μ_cr_* is determined by solving the so-called eigenvalue problem (linear elastic stability theory). The LTB critical moment *M_cr_* is obtained as the product of the critical load multiplier *μ_cr_* and the maximum bending moment *M_y,max_*. In the analysis of beam critical resistance with the LTBeam program, the following can be taken into account: (a) beams with mono- or bisymmetrical cross section, (b) elastic, from the LTB standpoint, conditions of beam fixity in the support nodes, and also along the beam length, (c) different loading schemes, (d) any ordinates of the points of transverse load application with respect to the shear centre of the beam cross section. Additionally, the later software version (LTBeamN) makes it possible to account for the effect of the elastic fixity of the beam in the plane of its bending (i.e., with respect to the major axis of the cross section).

In the verification calculations, the predetermined values of fixity indices *κ_ω_* and *κ_ν_* (in the case of bilaterally fixed beams, index *κ_ν_* = 1) were adopted. The rigidity of elastic restraint against warping (*α_ω_*) and the rigidity of elastic restraint against rotation in the beam bending plane (*α_ν_*), which are necessary for computations with the use of the *LTBeam* and LTBeamN program, were calculated from Equation (5).

A comparative analysis involved steel beams (*E* = 210 GPa, *G* = 81 GPa) made from IPE300, HEA300, and HEB300 sections, with spans of *L* = 5 and 7 m, and beams made from IPE500, HEA500, and HEB500 sections, with spans of *L* = 8 and 10 m. The calculations took into account loads in accordance with the schemes shown in Table 3. Transverse loads were applied to the top flange (TF, *z_g_* = +*h*/2), to the axis of gravity of the cross section (CG, *z_g_* = 0), and to the bottom flange (BF, *z_g_* = -*h*/2), respectively. The analyses were performed for a full variation range of the index of fixity against warping *κ_ω_* (from 0 to 1).

### 5.2. The Case of a Bilaterally Fixed Beam (κ_ν_ = 1)

Table 5 lists exemplary results of calculations produced for an IPE300 beam with a span of *L* = 5 m under the load of: (a) a concentrated force at the midspan, (b) a uniformly distributed load, and c) a triangularly distributed load. Calculations were performed for a full variation range of the index of fixity against warping *κ_ω_* (from 0 to 1), in particular for: *κ_ω_* = {0, 0.25, 0.5, 0.75, 0.9, 1}. Critical moments *M_cr,u_* were determined through the M_LTB,EL,u_ software (see Section 3.2) and calculated with Equation (11) and the coefficients listed in Table 4. The resulting LTB critical moments of beams were compared with the results provided by the LTBeam software [63] (FEM), adopted as reference values.

Critical moments of LTB *M_cr_* (Table 5) determined with M_LTB,ELu_ software, compared with those resulting from FEM (LTBeam), differed by −0.2% to + 1.2% (column VII). When the approximate Equation (11) was used, the resulting differences ranged from –0.1% to +5.8% (column IX). Maximum differences were found for a triangularly distributed load. That resulted from the asymmetric load causing a slightly asymmetric LTB mode. It should be remembered that Equation (11) was developed assuming a symmetric mode of the loss of stability (relative to the beam midspan).

### 5.3. The Case of a Bilaterally Elastically Restrained Beam ($0\leq \kappa_{v}\leq 1$)

Comparative analyses were performed for a full variation range of the index of fixity against warping *κ_ω_* (from 0 to 1), and against rotation in the beam bending plane, with *κ_ν_* ranging from 0 to 1. The calculations were made for various combinations of values of the *κ_ω_* and *κ_ν_* indices. As a result, for the case of a concentrated force load or a uniform load, the following values were selected: *κ_i_* = {0, 0.2, 0.4, 0.6, 0.8, 1}*_i=ω,v_.* However, for a triangularly distributed load, the following were adopted: *κ_ω_* = {0, 0.2, 0.4, 0.6, 0.8, 1}, and *κ_v_* = {0, 0.2, 0.4, 0.564, 0.8, 1}. In this case, the introduction of the value of *κ_v_* = 0.564 instead of 0.6 resulted from a change in the occurrence of the maximum value of the moment *M_y_* (identified with *M_cr_*) for the very coordinate. That meant that the maximum *M_y_* occurred in the span for *κ_v_* < 0.564, whereas for *κ_v_* > 0.564 it was on the support under a greater load. As regards a uniform load, such a situation took place for *κ_v_* = 0.6, while for a concentrated force load with *κ_v_* < 1, the maximum value of *M_y_* always occurred in the span (for *κ_v_* = 1, the absolute values for the span and the support moment were equal). 

For each of the analysed beams, the LTB critical moment was estimated using Equation (13). The results produced were compared with the values obtained from the LTBeamN program (FEM).

Table 6 lists exemplary results of calculations produced for an IPE300 beam, with a span of *L* = 5 m, under the load of a force concentrated at the midspan, applied to the top flange (TF, *z_g_* = +*h*/2).

When compared with the LTBeamN results, the critical moments (Table 6) determined with the approximate Equation (13) differed by −3.1% to +3.2%.

Table 7 shows the results of calculations for selected combinations of *κ_ω_* and *κ_ν_*, for an IPE300 beam with a span of *L* = 5 m, under a uniform load and under a triangularly distributed load, at the height of the top flange.

A comparison of values in Table 7 indicates that the difference between critical moments determined by Equation (13) and those obtained through the LTBeamN program did not exceed ±5% in most cases.

The results in Table 6 and Table 7 can be used while testing the appropriate version of the approximate Equation (13) in the spreadsheet.

Table 8 collates maximum percentage differences between results calculated from Equation (13) for all the examined beams (see Section 5.1) and those obtained by the LTBeamN program (FEM). The greatest differences were found for a triangularly distributed load. As already mentioned, the reason lies in the asymmetry of load, which affects the accuracy of estimation of the moments *M_o_* and *M_u_*.

**Figure 5:**
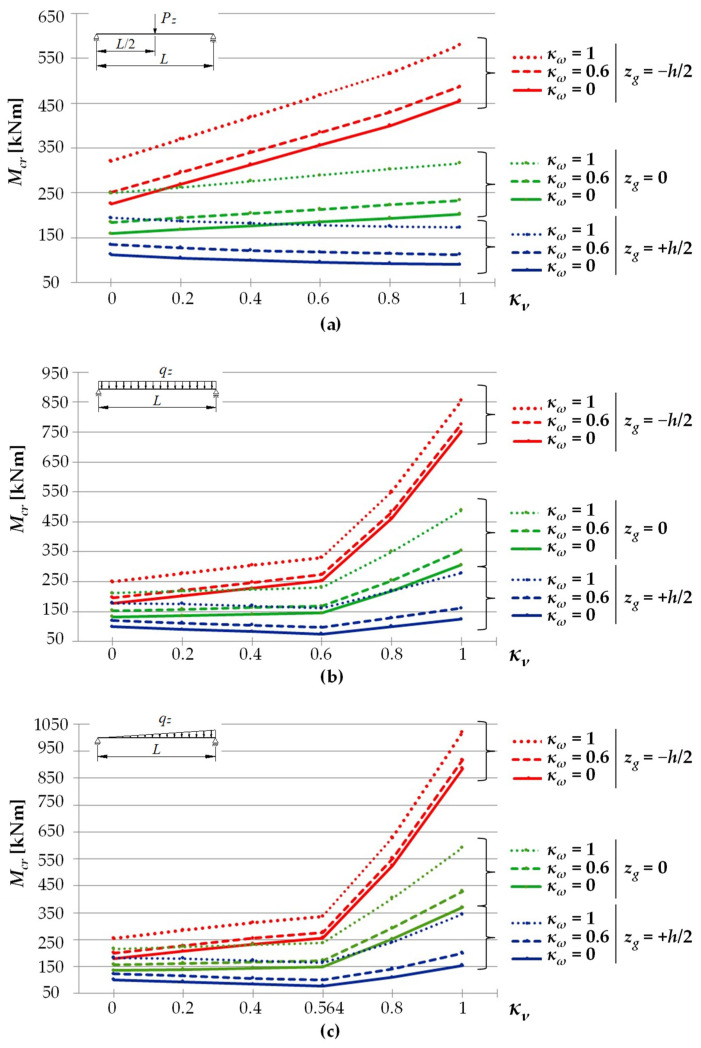
Critical moments of LTB *M_cr_* for an IPE300 (*L* = 5 m) beam as a function of the *κ_ν_* index for selected *κ_ω_* indices: (**a**) concentrated force load, (**b**) uniform load, (**c**) triangularly distributed load.

Figure 5 shows *M_cr_* variation trends for a beam made of an IPE300 section with a span of *L* = 5 m. *M_cr_* values vary depending on the values of the index of fixity against rotation in the beam bending plane, *κ_ν_* (from 0 to 1) for selected values of the index of fixity against warping, *κ_ω_* = {0, 0.6, 1}. A load took the form of: (a) a force concentrated at the beam midspan (Figure 5a), (b) a uniformly distributed load (Figure 5b), and (c) a triangularly distributed load (Figure 5c). The load was applied to the top flange (TF, *z_g_* = +*h*/2), in the axis of gravity (CG, *z_g_* = 0), or at the height of the beam bottom flange (BF, *z_g_* = −*h*/2). Critical moments were estimated by means of Equation (13).

A comparison of LTB critical moments of a beam under the load of a force concentrated at the midspan (Figure 5a) indicates that, for a load applied to the bottom flange (*z_g_* = −*h*/2), along with an increase in the *κ_ν_* index, a considerable increase (by an average of approx. +92%) in the values of *M_cr_* (red line) was observed. Basically, that happened regardless of the values of the *κ_ω_* index. For a load applied to the top flange (*z_g_* = +*h*/2), an increase in *κ_ν_* is accompanied by a fall (by an average of approx. −16%) in the values of *M_cr_* (the blue line). This results from a change in the moment *M_y_* distribution over the beam length as a function of the *κ_ν_* index. Obviously, this does not change the fact that, together with an increase in the value of *κ_ν_*, an increase in the value of the beam critical resistance (*P_cr_*) is observed. The analysed *M_cr_*(*κ_ν_*) relationships are practically linear over the entire variation range of the fixity index *κ_ν_* (from 0 to 1).

With respect to a beam under a uniform load (Figure 5b), generally bilinear *M_cr_*(*κ_ν_*) relationships were noted, with a kink in the curve for *κ_ν_* = 0.6 (the graph is slightly nonlinear only in the case of *z_g_* = −*h*/2 in a range of 0.6 < *κ_ν_* < 1). As already mentioned, for *κ_ν_* = 0.6, a change in the site of the maximum moment *M_y_* occurrence is observed. Within the variation range of the fixity index *κ_ν_* from 0 to 0.6, for a load applied to the bottom flange (*z_g_* = −*h*/2), an increase (by an average of approx. +38%) in the values of the critical moment (the red line) was found. However, for a load applied to the top flange (*z_g_* = +*h*/2), a fall (by an average of approx. −18%) in the moment *M_cr_* (the blue line) is seen. Like for the concentrated force load, the effect is caused by a change in the moment *M_y_* distribution over the beam length as a function of the *κ_ν_* index. Also in this case, the value of the beam critical resistance (*q_cr_*) grows with an increase in the values of *κ_ν_* (see Figure 4). However, in a range of 0.6 < *κ_ν_* < 1, an increase in the *κ_ν_* index is accompanied by an increase in *M_cr_* expressed as a function of the index of elastic restraint against warping *κ_ω_* (the largest one for *z_g_* = −*h*/2). When the rotation in the beam bending plane (*κ_ν_* = 1) was fully prevented, the resulting increase in *M_cr_* ranged from approx. +26% (*κ_ω_* = 0, *z_g_* = +*h*/2) to approx. +324% (*κ_ω_* = 0, *z_g_* = −*h*/2) compared with complete freedom of rotation (*κ_ν_* = 0).

With respect to a beam under a triangularly distributed load (Figure 5c), as was the case with a beam under a uniform load (see Figure 5b), the resulting *M_cr_*(*κ_ν_*) relationships were basically bilinear, with a kink in the curves at *κ_ν_* = 0.564. However, in this case, a more rapid increase in *M_cr_* was observed above this point. Within a range of variation of the fixity index *κ_ν_* from 0 to 0.564 (Figure 5c), for a load applied to the top flange (the blue line), a fall in the *M_cr_* values was noted. It was comparable to fall in the *M_cr_* values under the uniform load (cf. Figure 5b). When a load was applied to the bottom flange (the red line), an increase in *M_cr_* could be seen (Figure 5b). Full prevention of rotation in the beam bending plane (*κ_ν_* = 1), in contrast to its complete freedom (*κ_ν_* = 0), resulted in an increase in *M_cr_* from approx. +53% (*κ_ω_* = 0, *z_g_* = +*h*/2) to approx. +391% (*κ_ω_* = 0, *z_g_* = −*h*/2).

**Figure 6:**
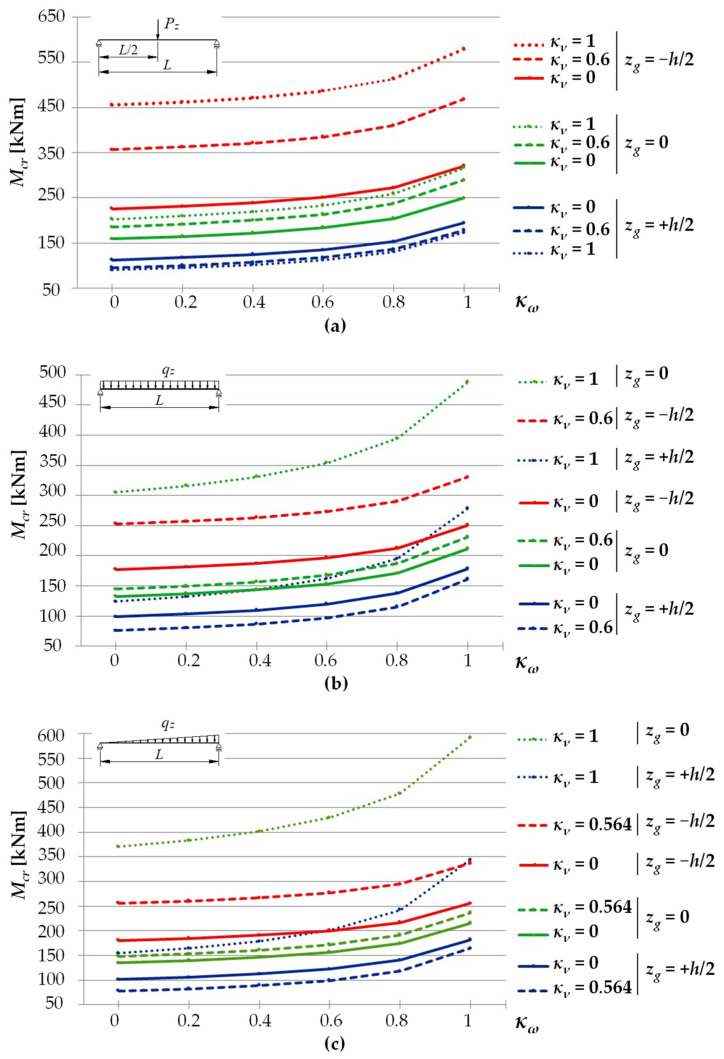
Critical moments of LTB for an IPE300 (*L* = 5 m) beam as a function of the *κ_ω_* index for selected values of the *κ_ν_* index: (**a**) concentrated force load, (**b**) uniform load, (**c**) triangular load.

Figure 6 shows the trends in variation in LTB critical moments *M_cr_* of the beam with geometrical parameters as per Figure 5. LTB critical moments varied depending on the values of the index of fixity against warping *κ_ω_* (from 0 to 1). In Figure 6a,b, the variation was shown for selected values of the index of fixity against rotation in the beam bending plane, *κ_ν_* = {0, 0.6, 1}, and in Figure 6c *κ_ν_* = {0, 0.564, 1}. Critical moments of the beam were estimated by Equation (13). The *M_cr_*(*κ_ω_*) relationships were slightly nonlinear over the entire variation range of the fixity index *κ_ω_* (from 0 to 1). It should be noted that, to increase the clarity of the *M_cr_*(*κ_ω_*) graphs, in Figure 6b,c it was decided not to show cases for *κ_ν_* = 1 at *z_g_* = −*h*/2, since their values greatly exceeded *M_cr_* for the remaining curves. In this case, for a uniform load, the *M_cr_* graph ranged from approx. 750 to approx. 860 kNm, and for a triangularly distributed load the range was from approx. 880 to approx. 1020 kNm.

**Figure 7:**
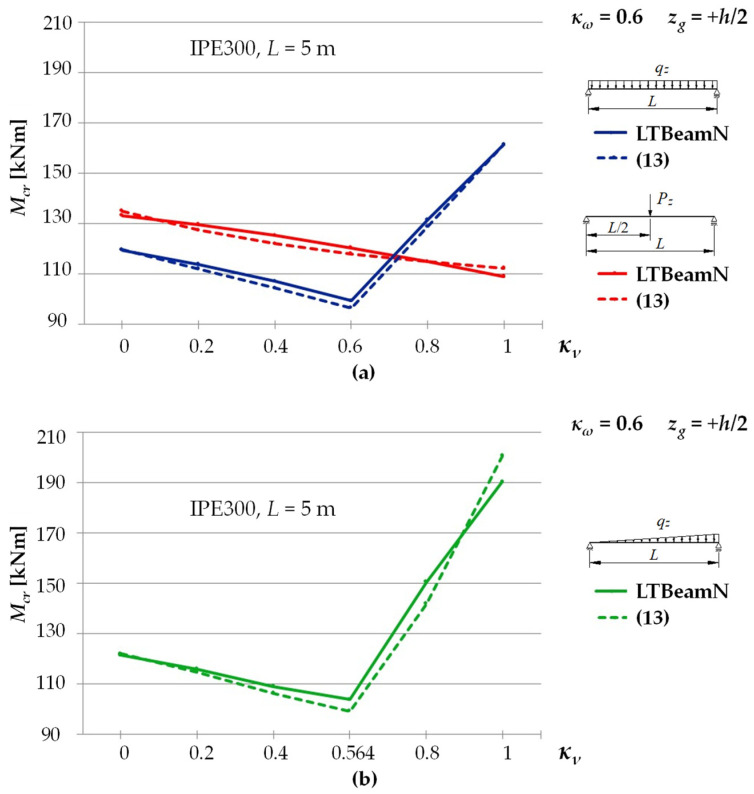
Comparison of graphs showing the variation in LTB critical moments *M_cr_*, determined by *LTBeamN* program and calculated with Equation (13): (**a**) a concentrated force load and a uniform load; (**b**) a triangularly distributed load.

Figure 7 shows a comparison of the variation trends in LTB critical moments *M_cr_* of the beam, determined by the LTBeamN program (the solid line) and calculated with Equation (13) (the broken line), depending on the index of fixity against rotation in the beam bending plane *κ_ν_* (from 0 to 1), for the index of fixity against warping *κ_ω_* = 0.6 and the ordinate of the applied load *z_g_* = +*h*/2.

The exemplary graphs of *M_cr_* variation (LTBeamN vs. Equation (13)) shown in Figure 7 have very similar shapes. The most noticeable discrepancy was found for a beam under a triangularly distributed load (see Figure 7b) for the fixity index *κ_ν_* ranging from 0.4 to 0.8 and for *κ_ν_* = 1. In that case, the maximum percentage differences in the results did not exceed ±6%.

### 5.4. Example of Calculations

The application of the approximate equations developed in the study is shown in the example. The procedure for solving the problem is illustrated by the consecutive steps of calculations.

Problem. A steel beam with an IPE 300 cross section (*I_y_* = 8360 cm^4^, *I_z_* = 604 cm^4^, *I_t_* = 20.7 cm^4^, *I_ω_* = 125,900 cm^6^) and a span of *L* = 6 m, is loaded by uniform forces applied to the top flange (*h*/2 = 15 cm). The rigidities of the beam elastic restraint in the support nodes are: (a) rigidity against warping: *α_ω_* = 27.9 kNm^3^/rad, (b) rigidity against rotation in the bending plane: *α_ν_* = 53,320 kNm/rad. Determine the LTB critical moment for the beam, *M_cr_*.

Solution.

1. Calculation of elastic restraint indices of the beam: (a) against warping according to Equation (1): *κ_ω_* = 0.76; (b) against rotation in the bending plane according to Equation (3): *κ_ν_* = 0.82;
2. The critical moment *M_o_* of a beam simply supported against bending relative to the axis of higher rigidity (*κ_ν_* = 0), and elastically restrained against warping (*κ_ω_* = 0.76), was calculated from Equation (11) and Table 3 (row 2): *M_o_* = 101.51 kNm;
3. The critical moment *M_u_* of a beam fully fixed against bending relative to the axis of higher rigidity (*κ_ν_* = 1), and elastically restrained against warping (*κ_ω_* = 0.76), was calculated from Equation (11) and Table 4 (row 2): *M_u_* = 146.73 kNm;
4. The coefficient of interaction *η* was calculated from Equation (18) for a range of 0.6 < *κ_ν_* < 1: *η*(*κ_ν_* = 0.82) = 0.395;
5. The LTB critical moment for an elastically restrained beam for the indices: *κ_ω_* = 0.76 and *κ_υ_* = 0.82 was calculated from Equation (13): *M_cr_ =* 101.51 + (146.73 – 101.51) ∗ 0.395 = 119.38 kNm.

When compared with the FEM calculations (LTBeamN): *M_cr,LTB_* = 118.95 kNm, the difference was 0.35%.

## 6. Conclusions

When taking into account the actual beam boundary conditions in the support nodes, which include elastic restraint against warping and elastic restraint against rotation in the beam bending plane, it is possible to calculate the critical moment precisely. Consequently, the LTB reduction factor and the beam design resistance can be determined more accurately. That points to the optimal design of this class of steel members.

Based on the results, it can be concluded that, from a technical standpoint, the LTB critical moments expressed as a function of the *κ_ω_* index, calculated with Equation (11) [52] and the coefficients in Table 3 (for a simply supported beam—*M_o_*), and the coefficients in Table 4, proposed in this study (for a bilaterally fixed beam—*M_u_*), provide sufficient approximation compared with FEM. Therefore, *M_cr_* for elastically restrained beams in the support nodes (*κ_ω_*, *κ_ν_*) can be estimated on the basis of Equation (13).

A comparison of critical moments (Table 6, Table 7 and Table 8), estimated by Equation (13) and obtained from the LTBeamN program (FEM), showed very good congruence with the results from an engineering standpoint. Critical loads were determined for: (1) different fixity indices (*κ_ω_*, *κ_ν_*) ranging from 0 to 1, (2) various (characteristic) points of transverse load application (top flange, axis of gravity of the cross section, bottom flange).

For a given value of the index of fixity against rotation in the beam bending plane (*κ_ν_*), an increase in the index of fixity against warping (*κ_ω_*) leads to an increase in the LTB critical moment. For example, for the beam shown in Figure 6a, for *κ_ω_* = 1, the increase in the critical moment ranged from approx. +27% (for *z_g_* = −*h*/2) to approx. +91% (for *z_g_* = *h*/2) compared with complete freedom of warping (*κ_ω_* = 0).

However, for a given value of the index of fixity against warping (*κ_ω_*), an increase in the index of fixity against rotation in the bending plane (*κ_ν_*) caused an increase in the beam critical resistance, measured by the respective critical load (*P_cr_*, *q_cr_, q_Tcr_*).

The effect of an apparent fall in the critical moment value, which occurs for beams under a load applied at the height of the top flange, resulted from changes in the distribution of *M_y_* expressed as a function of *κ_ν_* (see Figure 4). This effect was not observed when a load was applied at the cross section centre of gravity, or when the beams were under a load applied at the height of the bottom flange. 

For example, when the beam was under the load of a force concentrated at the midspan (Figure 5a), an increase in the values of *κ_ν_* at *z_g_* = −*h*/2 was accompanied by an increase in *M_cr_* (by an average of approx. 92%). That translated into an increase in the critical resistance *P_cr_* by an average of approx. 284%. Conversely, for *z_g_* = +*h*/2, in spite of a fall in the critical moment values (by an average of approx. 16% for *κ_ν_* =1), an increase in *P_cr_* by an average of approx. 68% was noted, compared with complete freedom of rotation (*κ_ν_* = 0). 

As regards a beam under a uniform load, for *κ_ν_* from 0.6 to 1 (Figure 5b), and a beam under a triangularly distributed load, for *κ_ν_* from 0.564 to 1 (Figure 5c), and any value of *κ_ω_* from a range of (0–1), the result showed a considerable increase in the values of *M_cr_* and critical load of the beam. However, within a variation range of the fixity index *κ_ν_* from 0 to 0.6 (Figure 5b) and from 0 to 0.564 (Figure 5c), an increase (by an average of approx. +38%, *z_g_* = −*h*/2) or a fall (by an average of approx. −18%, *z_g_* = +*h*/2) in the values of the critical moment was observed, compared with complete freedom of rotation (*κ_ν_* = 0). Also, for these beam loading schemes at *z_g_* = +*h*/2 (similar to the case of a concentrated force load), with the increase in *κ_ν_*, the beam critical resistance grew, in spite of the formal drop in the values of *M_cr_*. The effect can be seen in Figure 4. Complete prevention of rotation in the beam bending plane (*κ_ν_* = 1), compared with complete freedom of rotation (*κ_ν_* = 0), resulted in an increase in *M_cr_* from approx. +26% (*κ_ω_* = 0, *z_g_* = +*h*/2) to approx. +324% (*κ_ω_* = 0, *z_g_* = −*h*/2) (see Figure 5b), and from approx. +53% (*κ_ω_* = 0, *z_g_* = +*h*/2) to approx. +391% (*κ_ω_* = 0, *z_g_* = −*h*/2).

Taking into account a more precise description of the boundary conditions of beams with respect to fork support is a logical development in the modern design of steel structures.

Equations (11) and (13) can be applied to preliminary selection of the section and also, in many technically important cases, to principal design. The equations can also be used to verify *M_cr_* calculations with FEM software. Such verification will be helpful for less experienced designers. It can be employed to make necessary corrections, e.g., of errors in the specification of beam boundary conditions in the FEM programs. The major advantage of the adopted approach is that a steel structure reliability is already enhanced at the planning stage.

## Figures and Tables

**Table 1:** Application of polynomials.

Item	Polynomials	Physical Interpretation
I	II	III
1	$W_{P1}=\rho -2\rho^{3}+\rho^{4}$	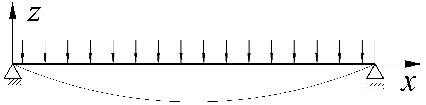
2	$W_{P2}=\rho -10\rho^{3}+15\rho^{4}-6\rho^{5}$	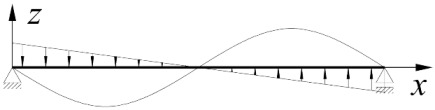
3	$W_{P3}=\rho -26\rho^{3}+73\rho^{4}-72\rho^{5}+24\rho^{6}$	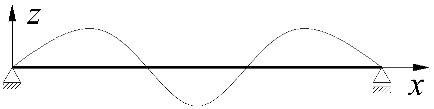
4	$W_{U1}=\rho^{2}-2\rho^{3}+\rho^{4}$	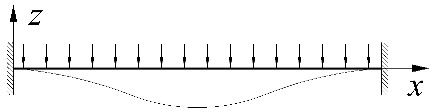
5	$W_{U2}=\rho^{2}-4\rho^{3}+5\rho^{4}-2\rho^{5}$	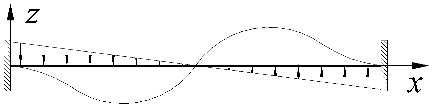
6	$W_{U3}=2\rho^{2}-13\rho^{3}+29\rho^{4}-27\rho^{5}+9\rho^{6}$	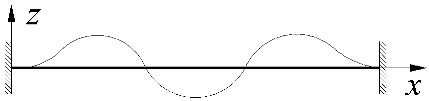

**Table 2:** Work done by external forces for selected static schemes.

Item	Static Scheme	The Work of External Forces
I	II	III
1	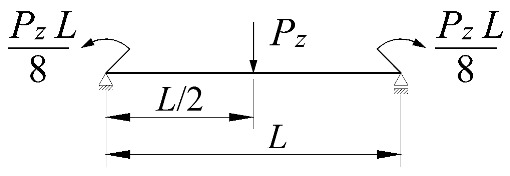	$T=\frac{P_{z}}{2}\left(\int_{0}^{L/2}\varphi \frac{d^{2}u}{dx^{2}}\left(x-\frac{L}{4}\right)dx+\right.$ $+\left.\int_{L/2}^{L}\varphi \frac{d^{2}u}{dx^{2}}\left(\frac{3L}{4}-x\right)dx+z_{g}\varphi_{L/2}^{2}\right)$
2	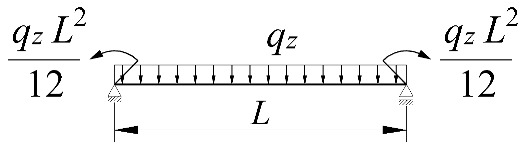	$T=\frac{q_{z}}{2}\left(\int_{0}^{L}\varphi \frac{d^{2}u}{dx^{2}}\left(L-\frac{L^{2}}{6x}-x\right)xdx+z_{g}\int_{0}^{L}\varphi^{2}dx\right)$
3	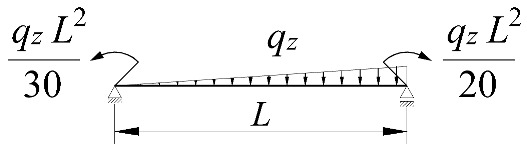	$T=\frac{q_{z}}{2}\left(\int_{0}^{L}\varphi \frac{d^{2}u}{dx^{2}}\left(\frac{3L}{10}-\frac{L^{2}}{15x}-\frac{x^{2}}{3L}\right)xdx+\frac{z_{g}}{2}\int_{0}^{L}\varphi^{2}dx\right)$

**Table 3:** Coefficients *B*_1_, *B*_2_, *B*_3_, *B*_4_ for simply supported beams (*M_cr,o_*) and selected loading schemes.

Item	Static Scheme	Coefficients
I	II	III
1	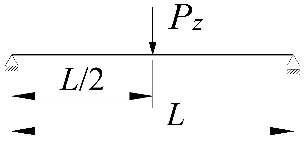	$B_{1}=7.242\cdot \left(1.563-2.5\kappa_{\omega }+\kappa_{\omega }^{2}\right)$ $B_{2}=1.522-2.467\kappa_{\omega }+\kappa_{\omega }^{2}$ $B_{3}=19.248\cdot B_{2}\cdot \left(1.457-2.4\kappa_{\omega }+\kappa_{\omega }^{2}\right)$ $B_{4}=231.816\cdot B_{2}\cdot \left(1.2-\kappa_{\omega }\right)$
2	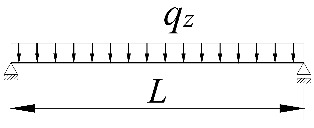	$B_{1}=5.250\cdot \left(1.476-2.429\kappa_{\omega }+\kappa_{\omega }^{2}\right)$ $B_{2}=1.507-2.455\kappa_{\omega }+\kappa_{\omega }^{2}$ $B_{3}=13.092\cdot B_{2}\cdot \left(1.457-2.4\kappa_{\omega }+\kappa_{\omega }^{2}\right)$ $B_{4}=157.633\cdot B_{2}\cdot \left(1.2-\kappa_{\omega }\right)$
3	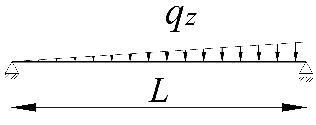	$B_{1}=5.322\cdot \left(1.476-2.429\kappa_{\omega }+\kappa_{\omega }^{2}\right)$ $B_{2}=1.507-2.455\kappa_{\omega }+\kappa_{\omega }^{2}$ $B_{3}=13.624\cdot B_{2}\cdot \left(1.457-2.4\kappa_{\omega }+\kappa_{\omega }^{2}\right)$ $B_{4}=163.486\cdot B_{2}\cdot \left(1.2-\kappa_{\omega }\right)$

**Table 4:** Coefficients *B*_1_, *B*_2_, *B*_3_, and *B*_4_ for selected static schemes of fixed beams (*M_cr,u_*).

Item	Static Scheme	Coefficients
I	II	III
1	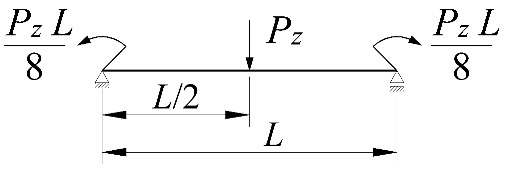	$B_{1}=23.333\cdot \left(1.563-2.5\kappa_{\omega }+\kappa_{\omega }^{2}\right)$ $B_{2}=1.522-2.467\kappa_{\omega }+\kappa_{\omega }^{2}$ $B_{3}=31.032\cdot B_{2}\cdot \left(1.457-2.4\kappa_{\omega }+\kappa_{\omega }^{2}\right)$ $B_{4}=372.934\cdot B_{2}\cdot \left(1.2-\kappa_{\omega }\right)$
2	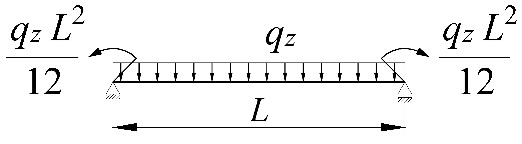	$B_{1}=42\cdot \left(1.476-2.429\kappa_{\omega }+\kappa_{\omega }^{2}\right)$ $B_{2}=1.507-2.455\kappa_{\omega }+\kappa_{\omega }^{2}$ $B_{3}=69.692\cdot B_{2}\cdot \left(1.457-2.4\kappa_{\omega }+\kappa_{\omega }^{2}\right)$ $B_{4}=839.664\cdot B_{2}\cdot \left(1.2-\kappa_{\omega }\right)$
3	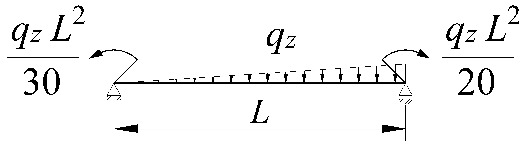	$\;B_{1}=49.033\cdot \left(1.476-2.429\kappa_{\omega }+\kappa_{\omega }^{2}\right)$ $B_{2}=1.507-2.455\kappa_{\omega }+\kappa_{\omega }^{2}$ $\;B_{3}=102.445\cdot B_{2}\cdot \left(1.457-2.4\kappa_{\omega }+\kappa_{\omega }^{2}\right)$ $B_{4}=1234.274\cdot B_{2}\cdot \left(1.2-\kappa_{\omega }\right)$

**Table 5:** Comparison of *M_cr,u_* for an IPE300 beam (*L* = 5 m) elastically restrained against warping.

Item	Static Scheme	*κ_ω_*	*z_g_*	*M_cr_* [kNm]				
				LTBeam	M_LTB,EL,u_	% VI–V	Equation (11)	% VIII–V
I	II	III	IV	V	VI	VII	VIII	IX
1	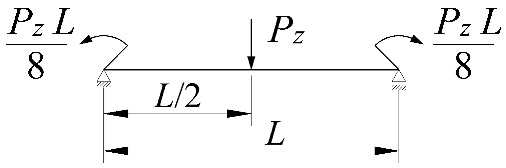	0	+*h*/2	87.7	88.4	0.7	90.2	2.8
2			0	201.0	201.3	0.2	202.6	0.8
3			−*h*/2	451.2	453.3	0.5	454.9	0.8
4		0.25	+*h*/2	93.8	94.5	0.8	96.5	2.9
5			0	209.7	210.1	0.2	211.5	0.9
6			−*h*/2	459.8	461.9	0.4	463.6	0.8
7		0.5	+*h*/2	103.4	104.3	0.8	106.5	2.9
8			0	223.4	223.8	0.2	225.5	1.0
9			−*h*/2	473.8	475.8	0.4	477.8	0.8
10		0.75	+*h*/2	121.4	122.5	0.9	125.1	3.0
11			0	248.4	249.0	0.2	251.3	1.2
12			−*h*/2	499.9	501.8	0.4	504.9	1.0
13		0.9	+*h*/2	142.4	143.8	1.0	146.9	3.1
14			0	276.8	277.5	0.3	281.1	1.6
15			−*h*/2	530.4	532.3	0.4	538.0	1.4
16		1	+*h*/2	167.4	169.3	1.2	172.8	3.2
17			0	309.6	310.7	0.3	316.4	2.2
18			−*h*/2	566.2	567.9	0.3	579.6	2.4
19	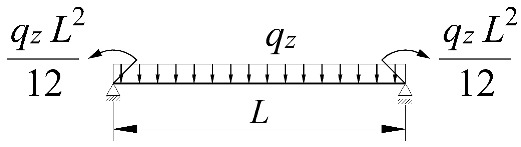	0	+*h*/2	124.4	124.4	0	124.2	−0.1
20			0	304.3	304.4	0	305.3	0.3
21			−*h*/2	727.9	732.8	0.7	750.3	3.1
22		0.25	+*h*/2	134.6	134.6	0	134.4	−0.1
23			0	317.9	318.0	0	319.1	0.4
24			−*h*/2	734.8	739.4	0.6	757.3	3.1
25		0.5	+*h*/2	151.3	151.3	0	151.3	0
26			0	339.4	339.6	0	341.1	0.5
27			−*h*/2	745.9	750.1	0.6	768.8	3.1
28		0.75	+*h*/2	183.9	183.9	0	184.3	0.2
29			0	379.1	379.3	0.1	381.9	0.7
30			−*h*/2	767.2	770.9	0.5	791.4	3.1
31		0.9	+*h*/2	224.1	224.1	0	225.4	0.6
32			0	424.6	425.0	0.1	429.8	1.2
33			−*h*/2	792.8	796.3	0.4	819.5	3.4
34		1	+*h*/2	274.3	274.3	0	278.3	1.5
35			0	478.1	478.6	0.1	488.1	2.1
36			−*h*/2	823.9	827.6	0.5	856.1	3.9
37	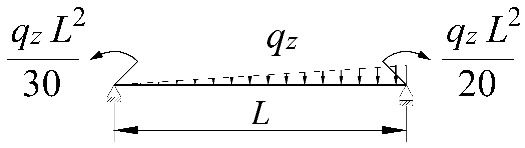		+*h*/2	147.2	148.7	1.0	154.7	5.1
38		0	0	359.7	359.9	0.1	370.1	2.9
39			−*h*/2	848.9	851.6	0.3	885.6	4.3
40			+*h*/2	159.2	160.8	1.0	167.3	5.1
41		0.25	0	375.9	376.1	0.1	386.9	2.9
42			−*h*/2	858.1	859.9	0.2	894.5	4.2
43			+*h*/2	179.0	180.8	1.0	188.1	5.1
44		0.5	0	401.4	401.7	0.1	413.5	3.0
45			−*h*/2	872.8	873.4	0.1	909.1	4.2
46			+*h*/2	217.5	219.6	1.0	228.7	5.2
47		0.75	0	448.4	448.8	0.1	463.0	3.3
48			−*h*/2	900.6	899.5	−0.1	937.4	4.1
49			+*h*/2	265.0	267.4	0.9	279.2	5.3
50		0.9	0	502.5	503.0	0.1	521.1	3.7
51			−*h*/2	933.3	931.3	−0.2	972.7	4.2
52			+*h*/2	324.9	327.3	0.7	343.9	5.8
53		1	0	566.1	566.8	0.1	591.8	4.6
54			−*h*/2	972.5	970.3	−0.2	1018.5	4.7

**Table 6:** Comparison of *M_cr_* for beam IPE300 (*L* = 5 m).

Item	Static Scheme	*κ_ω_*	*κ_ν_*	*M_cr_* [kNm]		
				LTBeamN	Equation (13)	% VI–V
I	II	III	IV	V	VI	VII
1	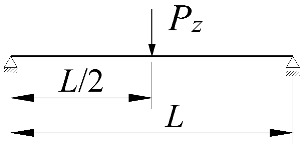 IPE300, *L* = 5m *z_g_* = +*h*/2	0	0	111.19	112.77	1.4
2			0.2	107.55	105.25	−2.1
3			0.4	103.13	99.89	−3.1
4			0.6	98.30	95.86	−2.5
5			0.8	93.08	92.73	−0.4
6			1	87.76	90.23	2.8
7		0.2	0	116.00	117.64	1.4
8			0.2	112.35	110.09	−2.0
9			0.4	108.00	104.70	−3.1
10			0.6	103.12	100.66	−2.4
11			0.8	97.82	97.51	−0.3
12			1	92.38	95.00	2.8
13		0.4	0	122.82	124.54	1.4
14			0.2	119.26	116.97	−1.9
15			0.4	114.93	111.56	−2.9
16			0.6	109.94	107.50	−2.2
17			0.8	104.58	104.35	−0.2
18			1	98.94	101.82	2.9
19		0.6	0	133.27	135.12	1.4
20			0.2	129.78	127.54	−1.7
21			0.4	125.50	122.13	−2.7
22			0.6	120.52	118.07	−2.0
23			0.8	115.02	114.91	−0.1
24			1	109.21	112.39	2.9
25		0.8	0	151.47	153.50	1.3
26			0.2	148.14	145.99	−1.4
27			0.4	143.90	140.64	−2.3
28			0.6	138.87	136.62	−1.6
29			0.8	133.08	133.49	0.3
30			1	127.12	130.99	3.0
31		1	0	191.80	194.13	1.2
32			0.2	188.74	187.01	−0.9
33			0.4	184.64	181.92	−1.5
34			0.6	179.58	178.10	−0.8
35			0.8	173.80	175.13	0.8
36			1	167.43	172.76	3.2

**Table 7:** Comparison of *M_cr_* for beam IPE300 (*L* = 5 m).

Item	Static Scheme	*κ_ω_*	*κ_ν_*	*M_cr_* [kNm]		
				LTBeamN	Equation (13)	% VI–V
I	II	III	IV	V	VI	VII
1	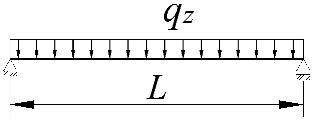 IPE300, *L* = 5m *z_g_* = +*h*/2	1	0	177.22	178.46	0.7
2		0.8	0.2	131.54	131.04	−0.4
3		0.6	0.4	107.24	104.49	−2.6
4		0.4	0.6	89.63	86.49	−3.5
5		0.2	0.8	109.05	106.12	−2.7
6		0	1	124.34	124.20	−0.1
7	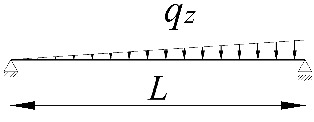 IPE300, *L* = 5m *z_g_* = +*h*/2	1	0	180.60	181.99	0.8
8		0.8	0.2	134.02	134.00	0
9		0.6	0.4	109.13	106.51	−2.4
10		0.4	0.564	93.77	89.01	−5.1
11		0.2	0.8	124.77	116.88	−6.3
12		0	1	146.70	154.68	5.4

**Table 8:** Summary of percentage differences of *M_cr_*.

Item	Static Scheme		Equation (13) vs. LTBeamN (%)
I	II	III	IV
1	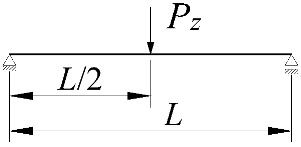	*z_g_* = +*h*/2	−3.7 ÷ 4.4
2		*z_g_* = 0	−1.7 ÷ 3.9
3		*z_g_* = −*h*/2	−2.4 ÷ 5.5
4	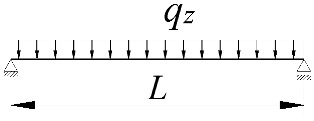	*z_g_* = +*h*/2	−5.2 ÷ 3.4
5		*z_g_* = 0	−0.6 ÷ 4.8
6		*z_g_* = −*h*/2	−6.0 ÷ 5.5
7	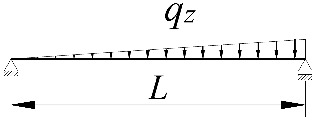	*z_g_* = +*h*/2	−7.1 ÷ 6.6
8		*z_g_* = 0	−1.4 ÷ 5.4
9		*z_g_* = −*h*/2	−6.1 ÷ 6.7
